# *Bordetella hinzii* in Rodents, Southeast Asia

**DOI:** 10.3201/eid1903.120987

**Published:** 2013-03

**Authors:** Tawisa Jiyipong, Serge Morand, Sathaporn Jittapalapong, Didier Raoult, Jean-Marc Rolain

**Affiliations:** Author affiliations: Aix-Marseille Université, Marseille, France (T. Jiyipong, D. Raoult, J.-M. Rolain);; Université Montpellier 2, Montpellier (S. Morand);; Kasetsart University, Bangkok, Thailand (T. Jiyipong, S. Jittapalapong); Kasetsart University, Nakhon Pathom, Thailand (T. Jiyipong);; Center of Excellence on Agricultural Biotechnology, Bangkok (T. Jiyipong)

**Keywords:** emerging zoonosis, zoonoses, Bordetella hinzii, bacteria, Southeast Asia, Cambodia, Laos, Thailand

**To the Editor:** Bacteria of the genus *Bordetella* are gram-negative, rod-shaped organisms that cause respiratory tract diseases in humans and animals. In 1995, *Bordetella hinzii* was isolated from poultry and 2 patients in the United States and France (*1*). This pathogen colonizes the respiratory tract of poultry and is closely related to *B. avium*, which is a commensal species in poultry. However, information on the etiologic role, hosts, and transmission routes of *B. hinzii* is incomplete because infections in human who did not have any close contact with poultry have been reported, mainly in immunocompromised patients (*1**–**5*). We obtained a single isolate of *B. hinzii* from blood agar culture during screening for bacterial zoonotic diseases in blood samples of rodents in Southeast Asia during the Ceropath project (www.ceropath.org).

During 2008–2010, we collected rodents along the Mekong River areas of 3 countries in Southeast Asia (Cambodia, Laos, and Thailand). Rodents were trapped in urban areas and in rural areas, which consisted of forests, upland and dry agricultural areas (orchards, cassava fields), unirrigated and irrigated agricultural areas (rice fields), and domestic areas (isolated farms and villages). Each animal was identified at the species level by using morphologic or molecular methods. Two hundred six blood samples were cultured on Columbia agar containing 5% sheep blood and incubated at 37°C for 3–7 days. A single atypical isolate was observed after 2 days of culture. This isolate was identified by using matrix-assisted laser desorption ionization time-of-flight mass spectrometry as described by Seng et al. (*6*). However, this isolate was identified only at the genus level as a *Bordetella* sp. (score 1.7).

To identify the *Bordetella* species, DNA from the isolate was extracted by using the QIAamp DNA Kit (QIAGEN, Hilden, Germany). Partial PCR amplification and sequencing of 16S rRNA gene was performed as described (*7*). Sequence analysis showed that the isolate was closely related to *B. hinzii* LMG 13501 (99.0% homology), which was isolated from the blood of a patient who died of septicemia in 2000 (*2*). The 16S rRNA sequence of our isolate (*B. hinzii* L135) has been deposited in GenBank under accession no. JX188059. A phylogenetic analysis of the new sequence and sequences of other bacteria in the genus *Bordetella* is shown in the Figure.

**Figure:**
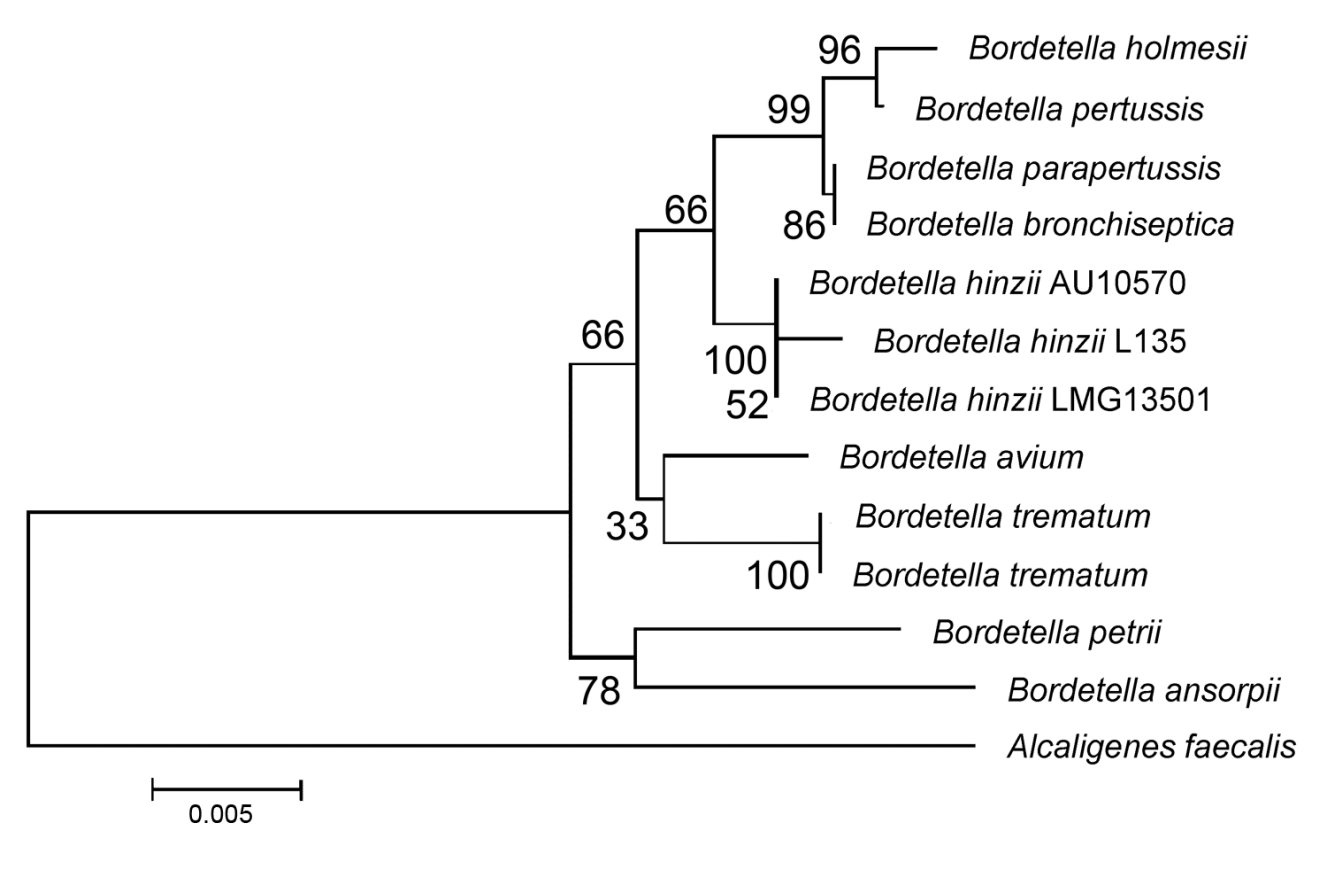
Maximum-parsimony phylogenetic tree of 16S rRNA gene of *Bordetella hinzii* isolate from this study (L135) and validated *Bordetella* species. Numbers along branches indicate bootstrap values. Scale bar indicates nucleotide substitutions per site.

*B. hinzii* is a causative agent of respiratory tract illnesses in birds and has been described as an emerging and opportunistic pathogen in immunocompromised patients; and in patients with AIDS, cystic fibrosis, and fatal septicemia (*1**–**5*). However, the source of transmission is not clear. Although *B. hinzii* is commensal in birds, several cases were reported in persons who did not have any close contact with birds (*2**–**5*), suggesting alternative sources of contamination. Thus, transmission routes and reservoirs of *B. hinzii* infection are ambiguous. *B. hinzii* infection has also been reported in rabbits and laboratory mice in Hungary and Japan (*8*–*10*). Rodents were suspected to be potential reservoirs but, to the best of our knowledge, this emerging pathogen has not been reported in wild rodents.

We detected in *B. hinzii* in a *Rattus tanezumi* rat that was trapped in upland agricultural area in Laos. *R. tanezumi* rats are the most common rodent in southeastern Asia and can be found in various habitats, including forests, agricultural areas, and houses. In Southeast Asia, human populations in several countries (Cambodia, Laos, and Thailand) live in close contact with rodents (including *R. tanezumi*) or share the environment with them. These rodents are known to be a reservoir and possible source of bacterial zoonoses such as leptospirosis, plague, scrub typhus, and murine typhus.

Our findings suggest that *B. hinzii* isolated from wild rodents may serve as reservoir for this bacterial species that could be transmitted to human or pets. *B. hinzii* should be added to the list of emerging bacterial zoonotic agents in wild rodents that could be pathogenic for humans. Further studies are warranted to evaluate the prevalence of this bacterium in rodents in other countries and to demonstrate that rodents may be a source of transmission of this bacterium to humans, especially immunocompromised patients.
